# Cerium; Crystal Structure and Position in The Periodic Table

**DOI:** 10.1038/srep06398

**Published:** 2014-09-17

**Authors:** Börje Johansson, Wei Luo, Sa Li, Rajeev Ahuja

**Affiliations:** 1 Applied Materials Physics, Department of Materials Science and Engineering, Royal Institute of Technology, SE-100 44 Stockholm, Sweden; 2 Condensed Matter Theory Group, Department of Physics and Astronomy, Uppsala University, Box 516, SE-751 20 Uppsala, Sweden

## Abstract

The properties of the cerium metal have intrigued physicists and chemists for many decades. In particular a lot of attention has been directed towards its high pressure behavior, where an isostructural volume collapse (γ phase → α phase) has been observed. Two main models of the electronic aspect of this transformation have been proposed; one where the 4f electron undergoes a change from being localized into an itinerant metallic state, and one where the focus is on the interaction between the 4f electron and the conduction electrons, often referred to as the Kondo volume collapse model. However, over the years it has been repeatedly questioned whether the cerium collapse really is isostructural. Most recently, detailed experiments have been able to remove this worrisome uncertainty. Therefore the isostructural aspect of the α-γ transition has now to be seriously addressed in the theoretical modeling, something which has been very much neglected. A study of this fundamental characteristic of the cerium volume collapse is made in present paper and we show that the localized ⇌ delocalized 4f electron picture provides an adequate description of this unique behavior. This agreement makes it possible to suggest that an appropriate crossroad position for cerium in The Periodic Table.

Cerium is one of the most fascinating elements^1^ in the periodic table and continues to attract a lot of attention. An early explanation for its most remarkable α-γ volume collapse was that this transition originates from a valence change of cerium^1^ from a trivalent rare-earth like metal with one localized 4f electron, to a tetravalent metallic state, comparable to titanium or zirconium. The idea was that the localized 4f electron in trivalent cerium becomes promoted to the conduction band. This promotion model provides a satisfactory explanation of the drastic volume collapse, since four valence electrons bind considerably stronger than just three.

However, it was later demonstrated that the energy difference between these two metal valence states in cerium is large^2^, of the order of 2 eV, completely invalidating this conventional valence change picture for the α-γ transition. Similarly, when more sophisticated experimental tools were made available for studies at high pressure, it became increasingly clear that there is no dramatic change in the electron occupation of the 4f orbital across the volume collapse region^3^,^4^. Since then many spectroscopic investigations^5^,^6^,^7^ have been directed to the cerium problem.

Early on it was proposed that the pressure induced collapse was due to a fundamental change of the character the 4f state^2^. On the low density side the 4f electron is localized, forms an atomic-like magnetic state and does not contribute to the interatomic bonding. In the high density phase the 4f electron is instead itinerant, metallic, and contributes significantly to the bonding causing the volume collapse. This metallization of the 4f-electron means that this phase transformation can be viewed upon as a Mott transition regarding the 4f behavior. Somewhat later an alternative picture for the α-γ transition was suggested by Martin and Allen^8^. In their treatment they focus on the interaction between the 4f electron and the conduction electrons in terms of the Kondo model.

In the ongoing strong efforts^9^,^10^,^11^,^12^,^13^,^14^,^15^,^16^ to provide a theoretical basis for the α-γ transition in cerium, very little attention has been directed towards the fact that the volume collapse is *isostructural*. In most recent experiments^17^ it has now been shown that the volume collapse retains the fcc structure removing earlier doubts about this behavior^18^,^19^. This fact makes it natural to search for an electronic reason for the phase transformation. However, after an identification of such a possible mechanism, the next and equally important step for theory is to identify the necessary conditions that have to be fulfilled in order to account for the preservation of the crystal structure. Focusing on crystal structure has also the great advantage that this is a ground state property.

The understanding of the crystal structures of the elemental metals is now very well established^20^. Electronic structure calculations reproduce for example nicely the experimental crystallographic data for the d-transition metals. By means of so called canonical bands, focusing on for example d-orbitals^21^, this success can also be expressed in appealing physical terms. For example the crystal structure sequence for the 4d and 5d transition metals can be directly explained as originating from the gradual filling of the d-band as one proceeds through the d series. By extending the canonical band concept to the ferromagnetic 3d metals^22^, also their anomalous crystal structures can be explained from the same simple physical concept, i.e. the filling of the d-band. More recently canonical bands have been extended to f-band metals^23^,^24^, i.e. the lighter actinide metals, and again it provides an attractive platform for the understanding of the crystallographic behavior. Here it is the gradual filling of the f-band which is decisive for the actual crystal structure. To summarize the theoretical understanding that has emerged we conclude: for d-band metals the d-electrons are responsible for the crystal structure, while for the earlier actinide metals the f-band electrons determine the atomic arrangement. The canonical d band concept tells us that this is independent of if we are dealing with 3d, 4d, or 5d elements. In the same way the canonical f-band picture is the same irrespective of if we are considering 4f- or 5f-elements.

## Results

**Figure 1:**
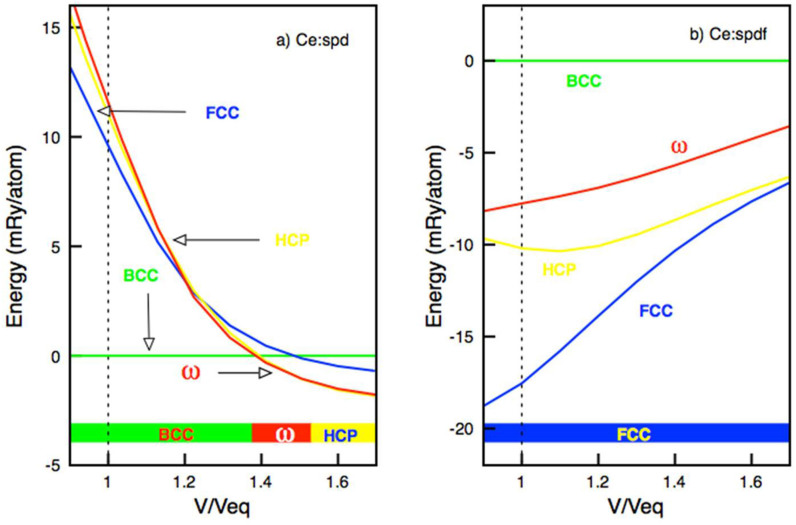
Calculated energy difference between the bcc, fcc, hcp, and ω crystal structures for Ce as a function of volume (V/V_eq_ with V_eq_ = 30.3 Å^3^) for the case when Ce is treated as a conventional tetravalent d transition metal (Fig. 1a, see text) and for the case when Ce is treated as an itinerant f-band metal, i.e. when the 4f states are included in the basis set (Fig. 1b). In both Fig. 1a and Fig. 1b the bcc structure is used as the zero energy reference level, and the dotted vertical line denotes the equilibrium volume.

Before using the canonical band concept, we perform some full-fledged electronic structure calculations for tetravalent transition metals as an illustration that crystal structure data indirectly can provide information about the electronic structure. We use the full-potential muffin-tin-orbital (FPLMTO) method^25^ and for the exchange and correlation potential the generalized gradient approximation (GGA)^26^ was applied. From both theory^27^ and experiments^28^,^29^ it has been established that the tetravalent 3d–5d transition elements under pressure exhibit the crystal structure sequence hcp → ω(AlB_2_) → bcc, a sequence originating from an increasing occupation of the d-states with pressure. The fact that cerium has four electrons in addition to the xenon core prompted us to study the mentioned typical tetravalent crystal structures also for this element, but now in addition including comparisons with the fcc structure. First we do this when only s, p and d orbitals are included in the basis set for the valence states and only thereafter we perform calculations where f orbitals have been added to the basis. Calculations with the restricted set of orbitals correspond to that tetravalent Ce is treated as a 3d–5d Group IVB element (Ti, Zr and Hf). The results from energy comparisons between the relevant crystal structures are shown in Fig. 1a and at the observed equilibrium volume for α-cerium (V/V_eq_ = 1) the bcc structure is strongly favored compared to the other structures. Thus the experimental fact that α-cerium adopts the fcc structure shows that a standard tetravalent (spd)^4^ description is not appropriate for this phase. However, at considerably expanded volumes the ω and hcp structures become favored and as a function of volume the same crystal structure sequence as observed for Ti, Zr and Hf is obtained, albeit here for Ce at negative pressures.

In Fig. 1b we show the results from a full calculation of the structural energy differences for tetravalent cerium as a function of volume. Thus the basis set now includes f orbitals in the theoretical treatment of the valence states. Here we find that at the equilibrium volume the fcc phase is energetically far more stable than the other structures. Thus the experimental fact that α-cerium adopts the fcc crystal structure demonstrates the importance of 4f electron participation in the bonding for this phase. In an additional full calculation for Ce we have put one electron into the 4f state and treated it as part of the inert core, i.e. not participating in the bonding. This is done in order to mimic trivalent γ-cerium. We find that also here the fcc structure is strongly favoured relative to the bcc structure (by 7 mRy/atom over the volume region of present interest ((28–37) Å^3^/atom)). This fits very well with the result from canonical d-band theory where the energy difference originates exclusively from d electrons.

**Figure 2:**
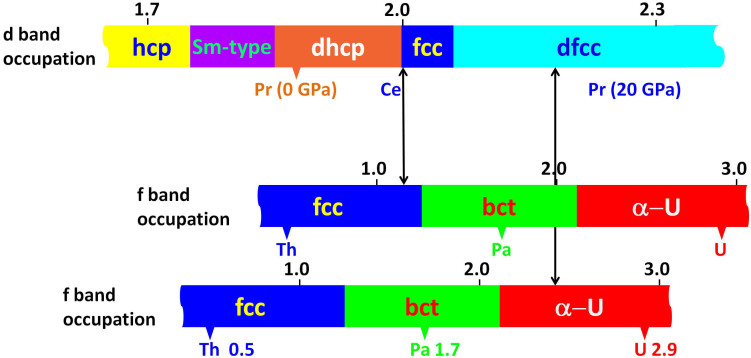
Illustration of the crystallographic changes which will take place when a light lanthanide metal (having d-electron bonding) under high pressure turns over into a state with f-electron bonding. For low pressure the local 4f^n^ magnetic moment remains stable and the crystal structure is determined by the occupation of the 5d band for the trivalent metal. This is the origin of the lanthanide structure sequence hcp, Sm-type, dhcp, fcc and dfcc′ (distorted fcc), illustrated in the topmost part of the figure. At high pressure, the local moment dissolves into a 4f band, and the crystal structure will now follow from the f band occupation number. In the figure the upper f-band occupation structure sequence is adopted to the appropriate f occupation for Ce (at the low volume for the phase change). The lower f-band occupation structure sequence is adjusted to the physical situation for Pr metal and therefore displaced relative to the case for Ce. Increasing pressure is to the right.

We now consider the low density side of the collapse in Ce (i.e. the γ-phase) and from now on we will base our treatment only on canonical bands. The construction of a generalized phase diagram for the trivalent lanthanide metals^30^ demonstrates the close interconnection between them. The very fact that such a diagram can be designed can also be seen as an independent confirmation of the insignificant role the 4f electrons play for the crystal structure in the trivalent lanthanide metals. Even the phase diagram for Ce in its low density crystal phases fits very well into this generalized phase diagram. This part therefore looks like it is leaning with a shoulder into the generalized phase diagram^31^ – while beyond this area Ce collapses into the high density phase region and no longer fits to the rest of the lanthanides. Thus the low density dhcp- and fcc-phases for Ce confirm the localized non-bonding property of the 4f^1^ configuration. Duthie and Pettifor^32^ used canonical d bands to explain the well-known structure sequence hcp → Sm-type → dhcp → fcc found for the trivalent lanthanides as one proceeds from the heavier elements towards the lighter ones or with pressure on an individual element. This pressure dependence is a direct consequence of the s to d electron transfer which occurs when the atomic volume is reduced^33^. From self-consistent calculations Skriver^34^ calculated the 5d occupation numbers for the trivalent lanthanide metals and provided values for when one structure turns into the next one. This dependence on the 5d occupation number for the lanthanide structure sequence is illustrated in the upper part of Fig. 2. Here the vertical (left) arrow shows the d-occupation appropriate for low density Ce at a compressed volume where the fcc structure is stable relative to the dhcp phase and close to the α-γ transition. In addition to this structure sequence, more recent experiments have shown that all the lighter lanthanides^35^, except cerium, at high pressure enters a distorted fcc structure (dfcc). Based on Skriver's calculations^34^ we have estimated an appropriate d-occupation value for the occurrence of this fcc-dfcc transition, and this has also been included in the structure sequence in the upper part of Fig. 2. The range of d-occupations shown in this figure covers all values relevant for the trivalent lanthanide metals and illustrates the evolution of the crystal structure as a function of the d-band occupation.

## Discussion

We now turn to the itinerant f-electron elemental metals (early actinide elements) and establish a similar diagram for the crystal structures of the 5f series as a function of the f-occupation, as we did above for the lanthanide d-elements. Here we meet rather exotic crystal structures so that the simple canonical band picture is not sufficient to account for the detailed behavior of the actinides. Söderlind^23^,^24^ has shown that by including Madelung energies for the structures - which is especially important for the distorted structures one encounters here - the canonical picture can be extended so that the structure sequence observed for the itinerant 5f-elements can be described. Thus for this series of elements it is the occupation of the 5f-band which governs the crystal structure. For simplicity we here use *experimental data* for the crystal structures to establish the appropriate structure sequence as a function of the 5f-band occupation. We also notice that for f band elements under compression there will take place a transfer of electrons from the ‘spd' bands into the 5f-band states, just like the s → d transfer for the d metals with pressure. In the case of thorium (fcc) it has been observed that this element transforms to the bct structure under compression around 100 GPa^36^. From our present calculations we find that for thorium the number of 5f electrons is about 0.5 at zero pressure, and that it has increased to 1.23 at the volume for the onset of the bct phase. With further compressions within the bct phase, the calculated f-occupation number approaches values similar to what it is at ambient conditions for the next element in the actinide series, protactinium. For this element we calculate that the 5f occupation is about 1.7 at zero pressure and the crystal structure is bct, which is indeed in perfect correspondence with the high pressure phase in thorium. Thus the behavior of thorium under high pressure can via the f-occupation be directly linked to Pa.

With pressure Pa has been found to transform to the α-uranium structure at 77(5) GPa^37^. At this transition we calculate an 5f occupation of 2.10. In the next element in the 5f series, i.e. uranium, one encounters the α-uranium crystal structure and at the equilibrium volume the f occupation for uranium is close to 2.9. From these data for the early actinide elements we arrive at a structure sequence fcc → bct → α-uranium as a function of increasing number of f-band electrons illustrated at the bottom of Fig. 2^38^ (This structure sequence can be related to the binary alloy phase diagram for adjacent actinide elements constructed in Ref. 38). For later use we also copy this structure sequence and plot it once more in the middle, but now slightly displaced from the first. Since the canonical band concept does not depend on whether this is for 4f- or 5f-orbitals, this structure sequence will be the same for those rare-earth metals where itinerant 4f-electrons are appropriate.

In the low density γ-phase of cerium there is *one* localized, non-bonding 4f-electron. This occupation number remains essentially the same in band calculations for the high density α-phase (n_4f_(α-Ce) = 1.15). We now connect the d occupation number for the γ phase to the f occupation number for the α phase, both at the α-γ transition. This is illustrated by a vertical arrow labeled Ce in Fig. 2. Thus we have connected the number for the d band occupation for γ-Ce (i.e. appropriate for the localized 4f phase, but close to the α-γ transition) with the corresponding appropriate occupation of the 4f band for α-Ce (delocalized 4f, again at the α-γ transition). Therefore we move from a d electron dominated crystallographic behavior, described by the top bar in Fig. 2, into an f band dominated electron crystal structure behavior illustrated by the middle bar in Fig. 2. This shows that for both cases (γ and α) we find an fcc crystal structure at the α-γ transition, exactly confirming the observed isostructural transformation behavior in Ce metal. Thus the present 4f delocalization model of the α-γ transition provides an appropriate description of the isostructural property of this phase transformation.

With increasing pressure the number of itinerant 4f electrons in α-Ce will increase. Experimentally one has identified the bct structure for cerium under high pressure, which is in full agreement with the structure dependence on the occupation of the f-band shown in Fig. 2 (lower part). The calculated f-band occupation in Ce is 1.22 for the volume where the transition to the bct phase is observed experimentally^39^ (We adopt the same effective bct structure representation of the initial high pressure phase in cerium as used in the reference 39). This practically coincides with the value for the 5f band occupation for the fcc-bct transition in thorium, demonstrating the adequacy of the concept of crystal structures governed by f-band occupation, and its independence of 4f- or 5f-bands. In addition, experimentally, depending on the sample treatment, one has also identified the α-uranium structure for cerium in this pressure region, a circumstance illustrating a sensitive competition between the bct and α-uranium structures.

The next trivalent element after cerium in the lanthanide series is praseodymium, which has a localized 4f^2^ electron configuration at equilibrium conditions. However, at a pressure of 20 GPa a volume collapse has been observed^40^,^41^. At such a pressure, but still within the localized 4f^2^ regime, the number of d electrons (n_5d_ (4f^2^ − Pr) = 2.24) is such that Pr is well within the distorted fcc region (Fig. 2 upper part). Now assuming the possibility of itinerant 4f-electrons we would for Pr expect a band occupation close to 2 at zero pressure, and we calculate it to be 2.43 at 20 GPa. Entering these numbers into Fig. 2 and connecting the topmost bar with the lowest one with a vertical arrow, we arrive at a crystal structure evolution of a similar type as above for Ce. From this figure we conclude that the volume collapse at 20 GPa in Pr should be from a distorted fcc structure into the α-uranium structure. This is in fact exactly what has been observed experimentally^34^. Just like in cerium we here meet another element where already knowledge about the crystal structure behavior heavily supports a 4f delocalization mechanism behind the volume collapse. This illustrates how crystal structure information can contribute to the understanding of the underlying electronic structure.

From Fig. 2 another interesting feature becomes apparent. Namely that the available region regarding the conditions for an isostructural fcc → fcc phase transformation is very limited. Thus it is only with a small margin that cerium is able to undergo this unique isostructural transition. From this figure it can also be concluded that there will be no other lanthanide element beyond cerium, that will display an isostructural volume collapse phase transformation. This is directly supported by the fact that already praseodymium, its neighbor element, does not show such a behavior, and instead its volume collapse is accompanied by a crystal structure transformation. However, it should be emphasized that it is not the crystallographic transition itself which is the driving force for the delocalization process in praseodymium, but rather the competition between the energy gained in the formation of a local moment and the energy gained from the establishment of metallic bonding.

**Figure 3:**
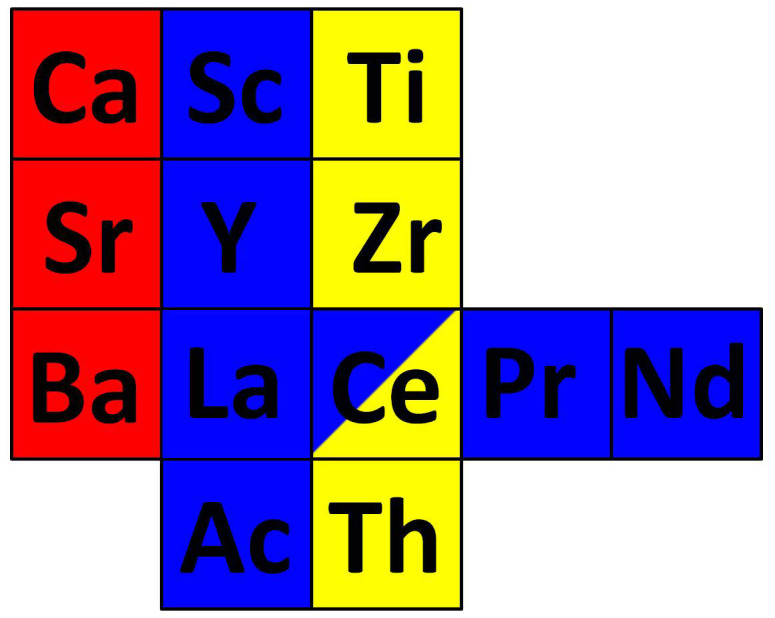
A generalized Periodic Table illustrating the dual role of cerium metal. In its γ phase cerium is best assigned as a member of the trivalent lanthanide series and in its α phase cerium is better described as a member of the Group IVB elements. Therefore the position of cerium in the Periodic Table should be in the crossroad between the lanthanide row and the Group IVB column.

A consequence of the findings above is the dual feature shown by cerium. In its γ-phase cerium is clearly best assigned as a member of the lanthanide series. However, in the α-phase cerium is better described as a member of the Group IVB elements. Its similarity to thorium, which also has itinerant f-electrons, is especially clear, where for example both elements appear in the fcc structure. Accordingly the position of the element cerium in the Periodic Table should be in the crossroad between the lanthanide row and the Group IVB column as illustrated in Fig. 3. In accordance with this, there are for example two oxides of cerium: the trivalent sesquioxide Ce_2_O_3_ and the tetravalent dioxide CeO_2_. In Ce_2_O_3_ the 4f-electron is localized and non-bonding, while in CeO_2_ the 4f-orbitals hybridize with the oxygen orbitals to contribute to the oxygen-cerium bond^42^. In this way the two oxides show a fundamental difference between a bonding and non-bonding influence of the 4f-electron, very much in accordance with the α-γ transition in cerium metal, when cerium is described as a Mott transition. In the present connection it is interesting to note that in the deoxidation (oxidation) process of CeO_2_ (Ce_2_O_3_) into Ce_2_O_3_ (CeO_2_) the Ce skeleton structure remains the same in both crystal phases^42^. In this sense this is a remarkable parallel case to the isostructural α-γ transition in cerium metal. As an illustrative example, this bonding to non-bonding transition of the 4f-electron is actually used in catalytic converters in automotive applications like minimization of CO emissions in the exhaust gases, involving a cycle process between Ce_2_O_3_ and CeO_2_. This illustrates that the concept of a Mott transition is of direct use in modern technology.

## Methods

We use the full-potential muffin-tin-orbital (FPLMTO) method^20^ and for the exchange and correlation potential the generalized gradient approximation (GGA)^21^ was applied.

## Author Contributions

B.J. designed research. W.L. performed research & made all figures. B.J. and R.A. wrote the paper & analyzed data. S.L. provides some tools.
